# Efficacy and safety of Shenmayizhi decoction as an adjuvant treatment for vascular dementia: Study protocol for a randomized controlled trial

**DOI:** 10.1097/MD.0000000000018326

**Published:** 2019-12-16

**Authors:** Huichan Wang, Nanyang Liu, Yun Wei, Hui Pei, Meixia Liu, XueMei Diao, Huiqin Zhang, Hao Li

**Affiliations:** a Graduate School, Beijing University of Chinese Medicine, Chaoyang District; b Department of Geriatrics, Xiyuan Hospital of China Academy of Chinese Medical Sciences, Haidian District, Beijing, China.

**Keywords:** clinical trials, Shenmayizhi decoction, traditional Chinese medicine, vascular dementia

## Abstract

**Background:**

Vascular dementia (VaD) is the second most common cause of dementia. The treatment of VaD still remains a challenge so far. Traditional Chinese Herbal medicine is a promising therapy due to their multiple components and targets. Shenmayizhi decoction (SMYZD), a Chinese Herbal prescription, has been reported its effective in alleviating cognitive dysfunction in clinical practice. However, strong clinical research of SMYZD in the treatment of VaD was lack. Therefore, we design this study to evaluate the adjuvant role of SMYZD in the treatment of VaD.

**Methods:**

This is a multicenter, randomized, blind, controlled trial. A total of 196 eligible patients will be assigned to receive Ginkgo biloba extracts (GBEs) plus SMYZD granule or GBEs plus SMYZD mimetic granule in a 1:1 ratio. The duration of the trial will be 12 weeks, and a follow-up will be performed at the 24th week. The primary outcomes are the National Institute of Health stroke scale (NIHSS) and the Alzheimer Disease Assessment Scale-cognitive subscale (ADAS-cog). The secondary outcomes include the Mini-Mental State Examination (MMSE), the traditional Chinese Medicine (TCM) syndrome scale, Activities of Daily Living (ADL), concentrations of hypersensitive C-reactive protein (Hs-CRP), neuron-specific enolase (NSE) and homocysteine (HCY) in serum. Researchers will record any adverse events throughout the trial.

**Discussion:**

This study will provide evidences to evaluate the efficacy and safety of SMYZD in combination with GBEs in treatment of VaD, as well as the adjuvant role of SMYZD in combination.

**Trial is registered at Chinese Clinical Trial Registry:**

ChiCTR1800017359.

## 1 Introduction

Vascular dementia (VaD) is the secondary cause of dementia after Alzheimer disease and accounts for 15% of all cases of dementia worldwide.^[1]^ In China, the prevalence of VaD is 1.50% for those aged over 65 years.^[2]^ Effective interventions or treatments to reduce the burden of VaD are urgently needed. However, no definitive medications or satisfactory pharmacological therapies have been introduced for the treatment of VaD. Currently, treatments are mainly focused on easing psychiatric symptoms or preventing cerebrovascular risk factors. Cholinesterase inhibitors and glutamate receptor antagonists have shown modest cognitive benefits in patients with VaD, yet they have not reached the expected therapeutic effects. In addition, their side effects are significant. Therefore, it is essential to develop and evaluate new promising treatments for VaD.

Traditional Chinese medicine (TCM) is drawing the researchers’ attention for its abundant experiences in the treatment of dementia. According to the theory of TCM, qi deficiency, blood stasis and hyperactivity of the liver yang are significant pathological factors of VaD. SMYZD is a compound formula with functions of supplementing qi, activating blood circulation, calming the liver and suppressing liver yang. Our preclinical pharmacological experiments have shown that SMYZD granule promoted neurovascular unit angiogenesis by enhancing hypoxia inducible factor 1 α, vascular endothelial growth factor and notch protein expression, and exerted anti-oxidative stress effects by regulating the Nrf2/HO-1 signaling pathway and anti-apoptotic effects though reducing the expression of caspase 3.^[3]^ SMYZD consists of four herbal drugs, including *Radix et rhizome Ginseng* (Ren Shen), *Rhizoma Gastrodiae* (Tian Ma), *Rhizoma Chuanxiong* (Chuan Xiong) and *Ramulus Euonymi* (Gui Jian Yu), the corresponding pharmacological targets of these herbs are listed in Table 1, which could be involved in the management of VaD.

**Table 1:**
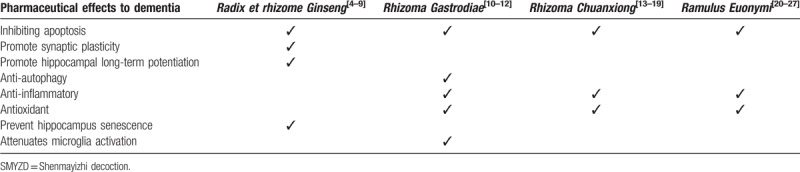
Multitarget mechanisms of SMYZD.

A pilot trial had been conducted to explore the efficacy of SMYZD, however, the pilot study was limited by the small sample size and absence of follow-up. The GBEs is believed to be effective and safe in the treatment Alzheimer disease and VaD.^[28,29]^ Therefore, the present study aims to evaluate the efficacy and safety of SMYZD as an adjuvant treatment for VaD using a randomized, controlled, blind and multicenter trial.

## 2 Methods

### 2.1 Study design

This study is a multicenter, randomized, blind, controlled trial with a 1:1 allocation. The present protocol was registered at Chinese Clinical Trial registry (Number: ChiCTR1800017359) on July 25, 2018, and we design this protocol in accordance with the Standard Protocol Items: Recommendation for Interventional Trials 2013.

**Figure 1:**
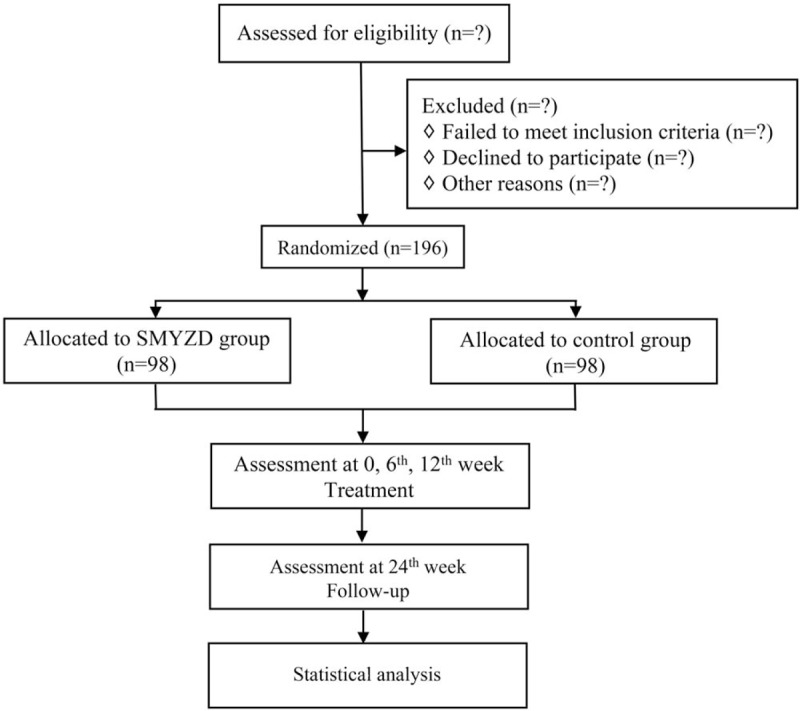
Flowchart of the study.

After a recruitment period and obtaining the signed informed consent from all the participants, eligible participants will be randomized to SMYZD group or control group, and receive treatment for 12 weeks, with a follow-up assessment at 24th week. A flowchart of the study is shown in Fig. 1. The evaluations and visits will be conducted according to the testing schedule as shown in Table 2.

**Table 2:**
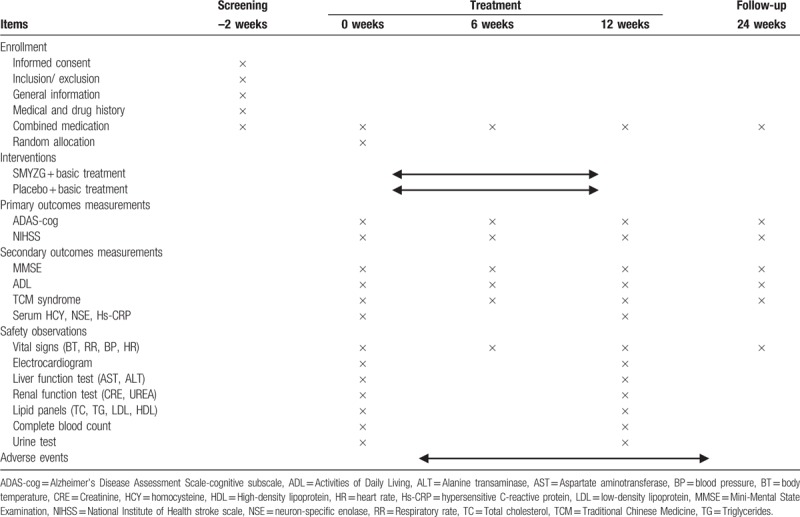
Schedule of the study process.

### 2.2 Ethics approval and consent to participates

The study protocol (version 1.1, date November 6, 2017) was approved by the ethics committees of the Xiyuan Hospital, China Academy of Chinese Medical Science (the sponsor of this study, approval number: 2017XLA033–4). The trial was registered at Chinese Clinical Trial Registry (Number: ChiCTR1800017359). The study's aims, procedures and possible side effects will be explained to the participants. Singed informed consent will be obtained from all the participants. No participants’ names or identifying information will be released.

### 2.3 Participants

#### 2.3.1 Inclusion criteria

Patients will be enrolled in this trial if they meet the following criteria:

(1) Patients 45≤ aged ≤ 75, male or female, and females should be post menopause;
(2) Clinical diagnosis of VaD according to the Diagnostic and Statistical Manual of Mental Disorders IV (DSM-IV)^[30]^ and NINDS-AIREN^[31]^;
(3) Clinical Dementia Rating (CDR) score of 1 to 2;
(4) MMSE score of 10 to 26;
(5) Hachinski Ischemic scale (HIS) score ≥7;
(6) Diagnosis of qi deficiency, blood stasis and hyperactivity of the liver yang syndrome according to Guiding Principle of Clinical Research on New Drugs of Traditional Chinese medicine (Trial).^[32]^
(7) Adequate intellectual function to provide voluntary signed informed consent.

#### 2.3.2 Exclusion criteria

Patients meet the following criteria will be excluded:

(1) Depression (Hamilton Depression Scale 17 score ≥7) or other mental disorders;
(2) Dementia caused by other diseases (eg, Alzheimer disease, epilepsy, encephalitis, Parkinson disease, trauma);
(3) Severe visual and hearing impairment, severe aphasia or limb dysfunction might affect assessment;
(4) Thyroid disease, vitamin deficiency or severe disease (severe heart, liver, renal system disease, cancer);
(5) Use of other drugs (including herbal medicine) that exhibit any known actions on the central nervous system within 3 months before baseline;
(6) Allergic to herbal medicine or Ginkgo biloba extracts;
(7) Currently participating in another clinical trial.

### 2.4 Recruitment

Participants will be recruited from Xiyuan hospital and community healthcare centers of Jingzhuang Town and Shen Jiaying Town, Yanqing District, Beijing. The enrollment will be advertised through hospital posters and oral promoting by researchers. The recruitment is now on-going. Eligible participants will sign the informed consent form and receive a copy one which contains the contact telephone of the investigator. A dedicated phone line will response inquiries about joining in the project (8 am to 6 pm, from Monday to Friday).

### 2.5 Sample size calculation

The sample size was calculated based on the mean change from baseline in the Alzheimer Disease Assessment Scale-cognitive subscale (ADAS-cog) score. The previous study show that 1.5-point could detect a difference on ADAS-cog,^[33]^ thus we set δ = 1.5. With α at 0.05, β = 0.2 (80% power), s = 3.5, and δ = 1.5. As a result, 85 participants were needed in each group. Considering a discontinuation of 15%, the sample size required for the study was 98 per group.

### 2.6 Randomization and blinding

Randomization will be stratified by study center, with allocation ratio of 1:1 for each group. A random list of numbers will be generated using SAS software (version 9.2) by an independent statistician at the Good Clinical Practice (GCP) institute of the Xiyuan Hospital, who is not involved in the data collection or analysis. Research drugs will be numerically labeled and packaged according to the random numbers by the statistician of GCP institute. Participants will be allocated randomly into SMYZD group or control group according to the visit sequence. Participants, caregivers, investigators, data collectors and outcome assessors in this trial will remain blinded to allocated intervention until the study completion. Unblinding is permissible under the following circumstance:

(1) Participants develop another severe disease that need to be treated urgently;
(2) Serious adverse event (SAE).

Withdrawal occurs when a participant's allocation or intervention is revealed.

### 2.7 Intervention

All of the participants will undergo a 12-week treatment. All participants will receive GBEs (1 tablet, 3 times daily) as the basic treatment. One tablet of GBEs contains 19.2 mg of flavonol glucoside and 4.8 mg terpine lactone. GBEs used in this study are produced by Yangtze River Pharmaceutical (group) Co., Ltd. (Stata Food and Drug Administration Approval No. Z20027949).

The SMYZD group will receive SMYZD granule (1 bag, twice daily) plus GBEs. One bag (2.4 g) of SMYZD granule. The control group will receive mimetic granule of SMYZD (1 bag, twice daily) plus GBEs. Mimetic granule consists of dextrin (95%) and SMYZD granule (5%), which matches SMYZD granule with size, shape, taste and package.

In order to assess the medication adherence, the unused granules and tablets will be required to return to the research team and recorded on the CRF. SMYZD and mimetic granules are provided by the Beijing Tcmages Pharmaceutical Co., Ltd. (Beijing, China)

### 2.8 Compliance, withdrawal and discontinuation

Several methods will be used to enhance the compliance and retention, including:

(1) all research drugs and scheduled assessments free of charge;
(2) feedback to the caregivers or participants about the results of each assessment via telephone;
(3) telephone reminder before each visit. The included participants can withdraw from the trial for any reason at any time.

Participants for the following reasons will be regarded as dropouts:

(1) loss to follow-up;
(2) poor compliance;
(3) voluntarily quit the project;
(4) using prohibited medications;
(5) urgency unblinding;
(6) development of another severe disease that needs to be treated during the study.

Participants will be instructed to stop using the study drugs and visit their doctor when they are in the situation of any undesirable effect. We will compensate participants for trial-related harms. The reasons of withdrawal will be recorded in detail and be analyzed at the end of the trial.

### 2.9 Prohibited concomitant medications

To promote comparability of study groups, drugs that recommended in the 2011 Chinese guidelines for the diagnosis and management of cognitive impairment and dementia (V): dementia therapy^[34]^ are prohibited, including:

1. cholinesterase inhibitors;
2. excitatory amino acid receptor antagonists;
3. antipsychotic medications;
4. long-acting benzodiazepines;
5. anti-depressive drugs;
6. anxiolytic drugs;
7. huperzine A;
8. drugs that regulate free radical metabolism;
9. Chinese medicines (including decoctions, herbal extracts, and Chinese patent medicines) that improve cognitive function.

Short-acting benzodiazepines will be restricted to use (less than 2 times per week, and it will be prohibited in the 72 hours before assessments). Other drugs that do not explicit any known actions on the central nervous system are permitted. The name, dosing period, frequency, dosing amounts of these drugs will be recorded on the CRF.

### 2.10 Outcome measurements

The following outcomes will be assessed by trained assessors at specific visit timepoint, as shown in Table 2.

#### 2.10.1 Primary outcomes

1. The changes in cognitive function will be assessed by Alzheimer Disease Assessment Scale-cognitive subscale (ADAS-cog).^[35]^ The total score of ADAS-cog ranges from 0 to 70, and a higher score indicates more severe dysfunction.
2. The neurological status will be evaluated by National Institute of Health stroke scale (NIHSS).^[36]^ The maximum score of NIHSS is 42, and a higher score represents more severe neurological deficit.

#### 2.10.2 Secondary outcomes

1. The Mini-Mental State Examination (MMSE) scale^[37]^ for assessing the extent of dementia.
2. The Activities of Daily Living (ADL)^[38]^ score for rating quality of life.
3. The changes in Traditional Chinese Medicine (TCM) symptoms will be assessed based on TCM syndrome scale score. The efficacy will be measured by a reduction (reduction = (before treatment score – after treatment score)/before treatment score × 100%) in the following scale: clinical cure: reduction ≧95%, markedly effective reduction ≧70%, effective: reduction ≧30%, invalid: reduction <30%.^[39]^
4. Serum concentration of hypersensitive C-reactive protein (Hs-CRP), homocysteine (HCY) and neuron-specific enolase (NSE). The morning fasting venous blood will be obtained from participants. Separated serum will be stored at −80°C for further test.

### 2.11 Safety measurements and assessments

Safety measurements (as described in Table 2) side effects and adverse events will be monitored and recorded, including event, date of onset, severity, duration, and relationship to the study drug. Serious adverse events will be reported to the ethics committee of Xiyuan hospital within 24 hours and followed until they are adequately resolved.

### 2.12 Data management and quality control

To protect the anonymity and privacy of participants, participants’ name or identifying information will remain anonymous. An independent organization will be responsible for the data management. Data will be imported into the database by 2 independent staffs. After confirming blindness, the database will be locked. Before this trial began, all the staffs will receive a standard training that covered the clinical trial process, the administration of the assessments, the case report form completion, and the use of study granules. Xiyuan Hospital, the supervisor, will audit the trial conduct of each clinical trial center at least once a month.

### 2.13 Statistical analysis

All the data will be analyzed by a statistician using SPSS 19.0 software (IBM Corp., Armonk, NY) in a blinded manner. Outcomes will be analyzed in accordance with the intention-to-treat (ITT) principle. Missing values will be handled by the multiple imputation method.^[40]^ Homogeneity of Baseline characteristics will be analyzed with *t* test for continuous variables and χ^2^ test for categorical variables, or with Wilcoxon Mann–Whitney test. Primary outcomes, and secondary outcomes will be analyzed by covariance with treatment groups as factors and baseline values as covariates. Mean difference will be used to illustrate the effect size.

## 3 Discussion

Vascular dementia is an enormous, worldwide public health problem. Traditional Chinese medicine is an important complementary and alternative medicine, which has been used for treatment of dementia-like symptoms for centuries. SMYZD, a Chinese herbal formula, was used as an adjuvant treatment of VaD in clinical practice in China. Preclinical studies support that SMYZD could intervene in complicated molecular pathways underlying the pathogenesis of VaD. The main aim of this study is to investigate the effect of SMYZD as an adjuvant role in the treatment of AD.

In the present study, besides using the ADAS-cog, MMSE, ADL and TCM symptom scales to assess the clinical curative effect on cognitive function, NIHSS will be introduced to evaluate neurological status. In addition, we monitor the serum concentration of Hs-CRP, NSE and HCY to explore the possible mechanism of SMYZD combined with GBEs on treating VaD.

This study also has some limitations. For example, this study is limited to 1 region (Beijing). It is unclear whether our findings will be representative of other regions of China.

In conclusion, findings of this study are expected to provide evidence for efficacy and safety of SMYZD as an adjuvant treatment of VaD.

## Acknowledgments

The authors are grateful for all the colleagues and co-workers from the Department of Xiyuan hospital, and the health center of Jingzhuang community and Shenjiaying community.

## Author contributions

**Conceptualization:** Yun Wei, Meixia Liu, Hao Li.

**Investigation:** Hui Pei, XueMei Diao, Huiqin Zhang.

**Supervision:** Hao Li.

**Writing – original draft:** Huichan Wang.

**Writing – review & editing:** Huichan Wang, Nanyang Liu.
